# Accuracy of high definition near infrared transillumination camera in detection of hidden proximal caries

**DOI:** 10.4317/jced.59413

**Published:** 2023-01-01

**Authors:** Amr Edrees, Olfat Hassanein, Omar Shaalan, Asmaa Yassen

**Affiliations:** 1 Doctoral Candidate, Department of Conservative Dentistry, Faculty of Dentistry, Cairo University; Assistant Lecturer, Department of Conservative and Esthetic Dentistry, Faculty of Dentistry, The British University in Egypt; 2 Professor, Department of Conservative Dentistry, Faculty of Dentistry, Cairo University; 3 Lecturer, Department of Conservative Dentistry, Faculty of Dentistry, Cairo University; 4 Professor, Department of Conservative Dentistry, Faculty of Dentistry, Cairo University; Department of Conservative and Esthetic Dentistry, Faculty of Dentistry, The British University in Egypt

## Abstract

**Background:**

Early caries detection became mandatory in modern dentistry. However, the traditional methods in caries detection had many limitations. Hence,a novel approach based on Near Infrared technology was introduced to overcome such limitations.

**Material and Methods:**

Proximal surfaces of 102 posterior teeth from 36 adult participants who fulfilled the eligibility criteria were assessed by two examiners using three diagnostic methods. Teeth were examined visually according to the criteria of the International Caries Detection and Assessment System (ICDAS-II) then examined by bitewing digital radiograph (BW) and near infrared light transillumination (NIRT) camera (Vista Proxi iX HD smart). Inter and intra observer agreements were assessed using Kappa test.Dignostic accuracy parameters and Area Under the ROC curve (AUROC) with 95% confidence interval (95% CI) were evaluated for the different caries assessment methods.

**Results:**

The results of inter-observer agreement showed an excellent agreement in the different groups. There was a statistically significant difference in the score distribution between ICDASII and VistaCam modalities (*P*-value <0.05). While there was no statistically significant difference in the score distribution between bitewing radiography and VistaCam modalities (*P*-value >0.05). ROC curve analysis revealed that VistaCam when compared with ICDASII had sensitivity (99.0%), specificity (50.0 %), diagnostic accuracy (98.0%) and Area under the ROC curve (AUC) was 0.745 with 95% Confidence Interval (0.649 – 0.826).When VistaCam compared with bitewing radiography, it showed sensitivity (100.0%); specificity (40.0%), diagnostic accuracy (97.1%) and AUC (0.700) with 95% confidence interval (0.601 – 0.787).

**Conclusions:**

NIRT based diagnostic modality is a promising method for detection of hidden proximal lesions overcoming the hazards of radiograph.

** Key words:**Bitewing radiography , ICDAS II , Near-infrared transillumination, proximal caries, VistaCam® iX Proxi.

## Introduction

Dental caries is one of the most prevalent clinical conditions globally. In Egypt its prevalence is about 60% according to the world health organization (WHO) survey in 2014. Current data from an epidemiological research on children, young adults and adults indicates a shift in caries incidence from the occlusal surfaces at younger ages to the proximal surfaces with advancing age (1). Also, the prevalence of proximal lesions was found to be high, even in low risk populations (2). The main goal of the modern dentistry is the early detection of incipient carious lesions. However, detection of incipient hidden proximal caries has always been problematic due to the lack of accessibility and visibility with its rapid rate of progression (3).

Visual inspection is considered as the basic and most commonly used method to detect caries in the dental clinics. It was found that 25%-40% of proximal caries lesions could not be detected by clinical visual examination only (4). So, temporary tooth separation (TTS) as an adjunct to visual examination was used by some clinicians for the detection of proximal caries (5). In addition, Bitewing radiography has been introduced as the standard method for the detection of proximal caries when direct visual examination is not possible. However, it was reported that 40–60% of tooth decalcification is required for the lesion to be radiographicaly detected. Also, it has many limitations as it did not show the lesions activity with having little but detecTable hazards of exposing individuals frequently to the ionizing radiations (6).

Near-infrared light transillumination (NILT) is a photo-optical technology used in caries detection by directing a light with long wavelength against the proximal surface of the tooth creating good contrast between healthy and carious tissue (7). Limited studies regarding the accuracy of the recently launched NIR proxi head of VistaCam iX HD system were available. Hence this clinical study was conducted. The null hypothesis tested is that there is no difference in reliability of the near-infrared imaging system (VistaCam iX Proxi) compared with digital radiography and ICDAS-II in detection of hidden proximal carious lesions.

## Material and Methods

The protocol of this diagnostic accuracy study was registered in www.clinicaltrials.gov with identification number NCT03923192. All procedures done in this study, involving human subjects, were approved by the ethical standards of the Research Ethics Committee of the Faculty of Dentistry, Cairo University (Approval no. CREC19629). This diagnostic clinical study was held in the outparticipant clinic of the Department of operative Dentistry, Faculty of Dentistry, The British University in Egypt, Egypt. Based on a study conducted by Baltacioglu *et al*. 2017 (4), Area under ROC curve for diagnostic accuracy of near-infrared imaging system was (0.785) and for Bitewing radiography, it was (0.630). By adopting an alpha (α) level of 0.05 (5%), power=80%. The predicted sample size (n) was a total of 102 teeth. Sample size calculation was done using MedCalc® 12.4.0 Software (Mariakerke,Belgium).

A convenient sample of 36 participants, 19 females and 17 males, were enrolled during regular dental screening after a detailed description for the research procedures and obtaining the informed consent. Participants were included when they had suspected proximal lesion(s) like dark shadow through mesial or distal marginal ridge with presence of adjacent. While, those had cavitated proximal lesions or periodontaly compromised teeth were excluded. The mean (SD) value for their age was (27.75±6.16) years. Number of teeth included in the study was (102) teeth; 60 upper (58.8%) and 42 lower teeth (41.2%).

Each lesion was examined by two calibrated examiners (AE and AY). Blinding was not applicable, but no information sharing was allowed between the examiners during study period. Also, each examiner was not allowed to read the case report during the re-diagnosing of the same case.

Both examiners have past experience in caries diagnosis using ICDAS-II criteria, digital radiographic interpretation and near-infrared transillumination detection camera. In addition, calibration sessions discussing the methodologies were scheduled for the two examiners two weeks prior to the start of the study. They examined 60 teeth from participants of the Restorative Dentistry Clinic. Then, they compared the results and monitored the differences in scores between them until full agreement was reached. All the participants were subjected to caries risk assessment using ADA Chart with excluding extreme high caries risk participants.

**Figure 1:**
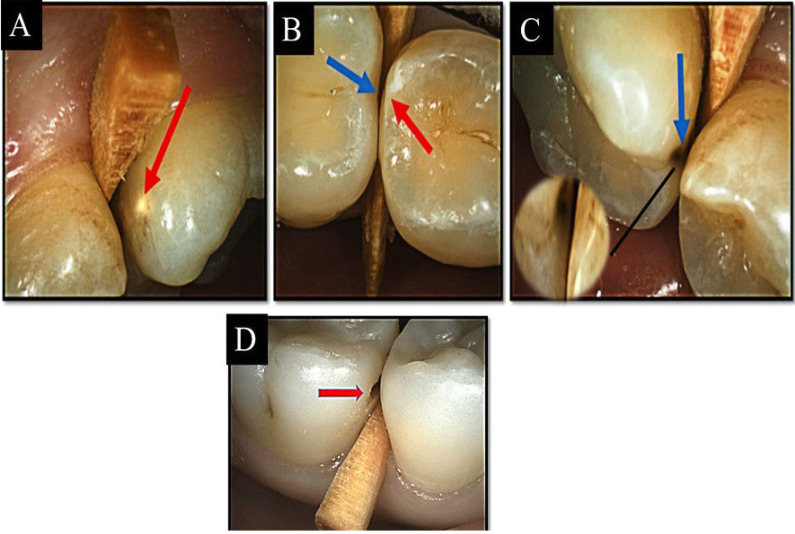
A-D) Intraoral images showing ICDAS-II scoring system from 0 – 4: A) Code 0 (red arrow); B) Code 1, 2 (red and blue arrow respectively); C) Code 3(blue arrow); D) Code 4 (red arrow).

Prior to the visual examinations, scaling for all the teeth was performed carefully using dental floss and a Water-Calcium Carbonate powder jet cleaner for 10 seconds and then rinsed for another 10 seconds using an air water spray in order to remove any remaining powder particles from the tooth surface. All visual examinations were performed under standardized light conditions from the dental unit with using a front-surface dental mirror, lightening system and an oil-free air syringe for drying teeth for 5 seconds and then isolated with cotton rolls. Teeth separation was done before the visual examination using wooden dental wedges sequentially until tooth separation occurs. After teeth separation, both examiners examined the proximal surface of the teeth visually in both wet and dry conditions. Then tactile examination was held using a dental mirror and a community periodontal index probe. Any signs indicating caries have been categorized according to ICDAS-II classification system (8) (Fig. 1). -Digital bitewing radiographic examination

After visual examination, bitewing digital radiographs of the examined teeth were taken with an intra-oral X-ray machine (Heliodent DS, Sirona, Bensheim, Germany) using A Phosphor Storage Plate and a 

RINN-set film holder to achieve comparable images with minimal overlap and standardization of images. The imaging plates were scanned using a phosphor plate scanner. The radiographic images were evaluated and scored from R0-R4 according to International Caries Classification and Management System (ICCMS) criteria (8) as follow: R0: no radiolucency, R1: radiolucency in the outer half of the enamel, R2: radiolucency in the inner half of the enamel with or without reaching the dentino-enamel junction, R3: radiolucency limited to the outer third of the dentin., R4: radiolucency reaching the middle third of the dentin.

-Near-infrared transillumination camera examination

**Figure 2:**
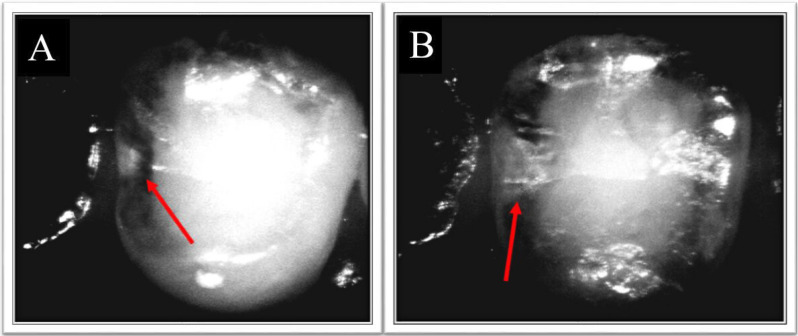
A,B) Near infrared transillumination image showing A) initial carious lesion extending in enamel (NR1) (Red arrow); B) carious lesion extending in dentin (NR2) (Red arrow).

After both visual and radiographic examination, the included teeth were imaged using VistaCam IX HD device with its proxi interchangeable head. The imaging was done in accordance with the manufacturer’s instructions after isolation using cotton rolls and suction tip and after tooth drying with a triplex air syringe for 15 seconds. The head of the intraoral camera was wrapped using a special hygienic protective cover provided by the manufacturer prior to imaging. The captured images were analyzed by using the DBSWIN software (Version 5.9.0) provided by the manufacturer. The extension of the lesions was assessed independently by the examiners and classified according to the following classification constructed according to the manufacturer’s information:0 = no signs of changes in enamel, NIR 1 = Wide bright strip or wedge-shaped structures within the dark translucent enamel ,NIR 2 = Wide bright strip or wedge-shaped structures, which seem to cross the enamel–dentin junction (Fig. 2).

As a result of the difference of the scoring system in each detection method, the resulted data were categorized as shown in  Table 1. This was done to allow homogeneity in the scores and allow better statistical analysis.

**Table 1:**
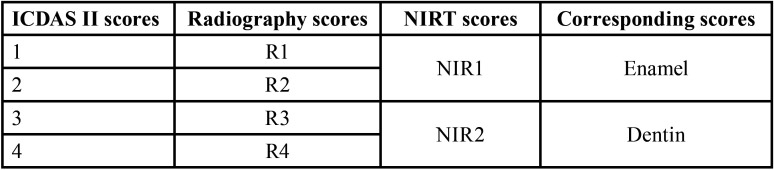
Different scores of each examination method and their corresponding scores in statistical analysis.

All the participants received dental treatment for the carious teeth, according to caries score and this was done for ethical, legal purposes and for participant`s benefit

-Statistical analysis

Statistical analysis was performed with IBM® SPSS® Statistics Version 26 for Windows. ROC (Receiver Operating Characteristic) curve was constructed to determine the diagnostic accuracy of (VistaCam) caries detection using ICDAS score and digital radiography as references using MedCalc Statistical Software Version 16.4.3. Inter and intra-observer reliability were analyzed using weighted kappa coefficient. Comparison between diagnostic modalities was done using Wilcoxon signed rank test. The significance level was set at *p* ≤ 0.05 for all tests with 95% confidence interval.

## Results

ROC curve analysis revealed that VistaCam when compared with ICDASII had sensitivity (99.0%), specificity (50.0 %), diagnostic accuracy (98.0%) and Area under the ROC curve (AUC) was 0.745 with 95% Confidence Interval (0.649 – 0.826).When VistaCam compared with bitewing radiography, it showed sensitivity (100.0%); specificity (40.0%), diagnostic accuracy (97.1%) and AUC (0.700) with 95% confidence interval (0.601 – 0.787) (Table 2).

**Table 2:**
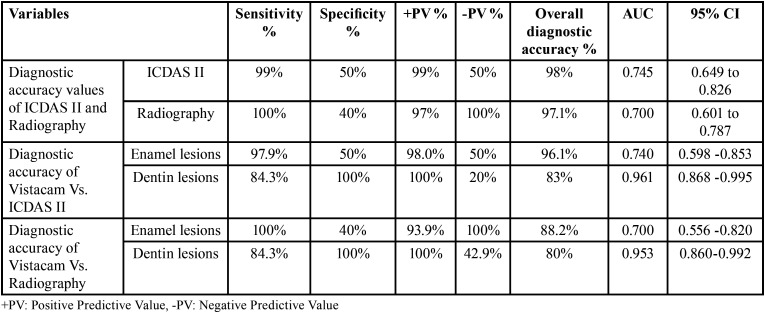
Overall and detailed diagnostic accuracy of near-infrared imaging system (VistaCam iX Proxi) with Visual inspection using ICDAS-II score and bitewing radiograph as references.

**Figure 3:**
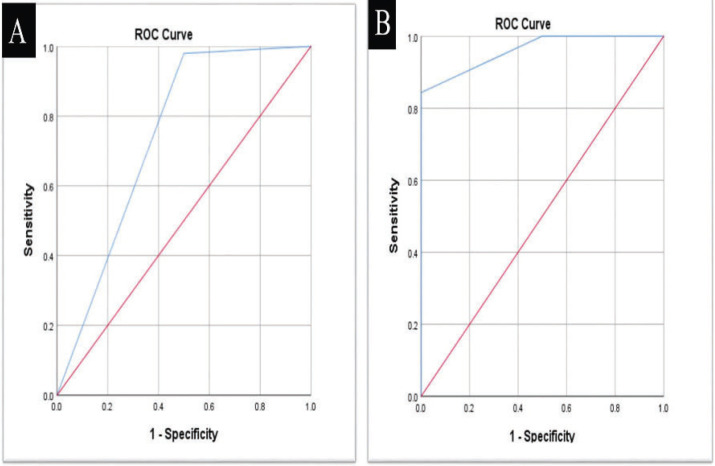
A,B) ROC curve between VistaCam with ICDAS II as a reference (A) enamel carious lesions; (B) dentin carious lesions.

The comparison between Vistacam and ICDAS II as reference showed that there was a statistically significant difference in the score distribution between both modalities (*p*=0.011). There was an equal prevalence of no caries detection in both modalities 2 (2.0%). Also, there was a higher prevalence of enamel caries detection in Vistacam 57(55.9%) in comparison to ICDAS 49(48.0%) while there was a higher prevalence of detection of caries in dentin in ICDAS II 51(50.0%) in comparison to Vistacam 43(42.1%).The results obtained from ROC curve analysis are showed in (Table 2, Fig. 3).

**Figure 4:**
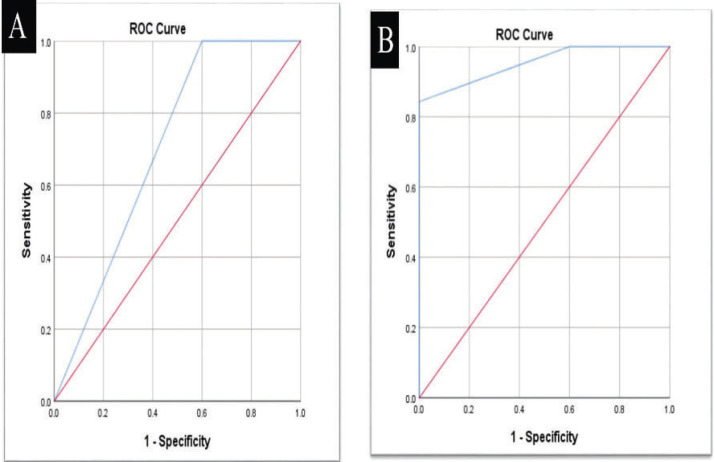
A,B) ROC curve between VistaCam with digital radiography as a reference (A) enamel carious lesions; (B) dentin carious lesions.

The comparison between Vistacam and digital radiography as a reference showed that there was no statistically significant difference in the score distribution in both modalities (*p*=0.132). Higher prevalence of enamel caries detection in VistaCam 57(55.9%) in comparison to digital radiography 46(45.1%) was detected, while there was a higher prevalence of no caries detection 5 (4.9%) and detection of caries in dentin 51(50.0%) in digital radiography in comparison to VistaCam 2 (2.0%) and 43(42.1%) respectively. The results obtained from ROC curve analysis are showed in (Table 2, Fig. 4). There was nearly perfect intra-observer agreement for ICDAS-II (Kappa= 0.942 for observer 1 and 0.873 for observer 2), for radiographic method (Kappa was 0.841 for observer 1 and 0.883 for observer 2) and for Vistacam method (Kappa was 0.880 for observer 1 and 0.855 for observer 2). Inter-observer agreement was nearly perfect for ICDAS-II, radiographic and VistaCam methods (Kappa= 0.942, 0.876, and 0.963, respectively).

## Discussion

Diagnosis of hidden proximal caries is difficult, where the presence of adjacent teeth prevents the direct visual and tactile examination. Proximal carious lesions were detected much earlier when visual-tactile examination is augmented by the teeth separation. Unfortunately, tooth separation may not always result in improved accessibility for direct examination of the proximal lesion and may also create patient discomfort. Therefore, tooth separation is not popular as a routine method for proximal caries detection, making an adjunctive method necessary. Bitewing radiography is known to be the most frequently used diagnostic aid in detection of hidden proximal caries. However, there are some concerns regarding the exposure of patients to ionizing radiation. Such limitations of the conventional methods for caries detection and the development of technologies led to the invention of modern optical devices for early caries detection. From these devices are the near infrared transillumination based devices. To the best of our knowledge, few studies were conducted to assess the the validity and accuracy of NIR VistaCam proxi in proximal caries detection especially with recent introduction of the high definition version (Vista Cam proxi Smart iX HD) of the device. Thus, this study was conducted to assess the reliability of the near-infrared imaging system using Vista Cam proxi iX HD in comparison with digital radiographic findings and visual examination using ICDAS-II in detection of proximal carious lesions.

The results of inter-observer agreement in the current study was calculated by Kappa test and they showed an excellent agreement in the different groups. These findings might be attributed to the calibration sessions which were done for both examiners regarding the optimal use of the three diagnostic methods. Moreover, standardization of the diagnostic process helped in this high agreement as the examiners used the same calibrated screen and lighting conditions for their assessments. Also, all values of the kappa coefficient for the intra-observer agreement showed perfect agreement. These results are in agreement with (7,9,10). On the other hand, Stratigiaki *et al*. 2020 (11) found that the intra- and inter-examiner reliability results showed a better performance of the bitewing radiography (BW) in comparison to NILT method. Such results might be attributed to the frequent use of radiographs making dentists being more familiar with the interpretation of the radiographic images and on the other hand they lacked the sufficient experience with NIRT methods.

Current results showed a statistical significant difference in the score distribution between Vista Cam proxi and ICDAS-II modalities. Furthermore, there was an equal prevalence of no caries detection in both modalities with a higher prevalence of enamel caries detection in Vista Cam proxi and a higher prevalence of caries detection in dentin using ICDAS II. These results might be because of the difference in lesion position as clear visualization of dentin caries with NIRT was only achievable if the lesion was located directly just beneath the occlusal enamel layer. While, the more cervically positioned dentin lesions with healthy dentin between the lesion and occlusal enamel could not be clearly observed as penetration of near-infrared waves decreased. Moreover, the extent of the caries and the degree of demineralization might affect the visualization. The present findings are in agreement with (12,13). While they disagreed with the results reported by Dezutter *et al*. (2020) (10) as they found that there was no significant differences between the NILT and ICDAS scores. This might be resulted from the lack of true status of the caries lesions evaluation which was considered as a prominent limitation of this study.

Another finding showed that Vista Cam proxi had an overall good level of agreement with ICDASII visual examination in different diagnostic measurements. Also, with reference to ICDAS II visual method, Vista Cam proxi had shown higher sensitivity in enamel with a great diagnostic accuracy more than its accuracy in dentin. On the contrary, the device showed low specificity in enamel when compared to that of dentin. The overall high sensitivity and low specificity of the Vista Cam proxi results in enamel surface might be referred to the difference in the near infrared radiations reflectance from the enamel surface giving high false positive results. Such difference could be resulted from either the difference in enamel thickness or size of contact areas. As increasing enamel thickness, will increase the amount of diffraction of near infrared light which will accordingly decrease the contrast between carious lesions and heathy thick enamel. Also, the surface curvatures and presence of either remaining stains or enamel cracks might affect the amount of refracted and reflected light giving false positive results in enamel (9,14,15). On the contrary, the overall low specificity of Vista Cam proxi in enamel might be referred to the high probability of light scattering from pores created as a result of preferential dissolution of the mineral in carious lesions. The relative low sensitivity of the Vista Cam proxi in dentin lesions could be attributed to the difficulty of distinction between the carious and healthy dentin as both are seen bright. Hence, the differentiation between different opacities in the resulted black and white image might be impossible in some cases (16).These findings are in agreement with (4,11,15,16,17,18). Also, there is an agreement with the systematic review and meta-analysis conducted by Marmaneu-Menero *et al*. (2020) (19) in terms of sensitivity and AUC values. On the other hand, such results are in disagreement with (7,20) which reported that NIRT method had high specificity and low sensitivity in proximal caries diagnosis. Such findings might be referred to the absence of true negative values as they were excluded leading to more spectrum bias. Also, Marmaneu-Menero *et al*. (2020) (19) reported high overall specificity of NIRT. This difference is a result of high heterogeneity of the meta-analysis in this review with lack of a specific gold standard and difference in devices used in each included study.

No statistically significant difference in the score distribution in both Vista Cam proxi and bitewing radiography (BW) modalities was found. There was a higher prevalence of enamel caries detection in Vista Cam proxi in comparison to digital radiography while there was a higher prevalence of no caries detection and detection of caries in dentin in digital radiography and in comparison, to Vista Cam proxi. These findings might be referred to the underestimation of enamel lesions by the digital radiograph as at least 30% of enamel demineralization is needed to be detected radiographically. Also, unlike radiography, NIRT does not have the issue of proximal surfaces overlapping, leading to detection of more enamel lesions (14). Increase number of readings in dentin lesions with radiograph than with Vista Cam proxi might be as a result of inadequate contrast between carious and sound dentin during the use of NIR light for transillumination. These results are in agreement with (4,9,12,13,21). While disagreement of these results was found with Kocak *et al*. (2020) (20). As they reported a significance difference in the enamel and dentin scores in both radiograph and Vista Cam proxi groups. This disagreement might be as a result of difference in reference standards in the study as visual tactile examination was the reference in enamel lesions while opening the cavities was the reference in dentin lesions which gave high difference in the resulted scores .

Another finding stated that Vista Cam proxi had an overall sufficient level of agreement with bitewing radiography in different diagnostic measurements. Moreover, with reference to the bitewing radiography, Vista Cam proxi had shown very high sensitivity with low specificity in enamel caries detection. While high sensitivity and high specificity in dentin caries detection with high overall diagnostic accuracy in all groups. However, slight decrease in sensitivity was reported in dentin when compared with enamel. This high overall sensitivity with low specificity, especially in enamel, might be attributed to the underestimation of initial enamel lesions by bitewing radiograph as it was accurate when the dentin was involved, but often led to the underestimation when only the enamel was affected (22). It is worth mentioning that, accurate sensitivity and/or specificity values cannot be determined just by comparing new technologies, such as NIRT, to older, unsatisfactory gold standards, such as intraoral radiography (21,23). As all the initial lesions were missed by radiograph and detected by Vista Cam proxi and these would be counted as false positive with the absence of true histological standard confirming their validity. Such increase in specificity of Vista Cam proxi in dentin when compared with radiograph might be attributed to the high accuracy of the radiograph in detection of dentin lesions .However, the slight fall in sensitivity value in dentin group might be as a result of the problem of contrast in images of Vista Cam proxi between dentin tissues. Also it might be resulted from deeper quantification of lesion depth which was seen with Vista Cam proxi compared to bitewing radiography (22). These results are in agreement with (11,16,24). While there is a disagreement with the results reported in a systematic review by Marmaneu-Menero *et al*. (2020) (19) as they reported low sensitivity of Vista Cam proxi. However, the reported specificity and AUC values came in agreement with our results. Such disagreement might resulted from the major variability in methodologies and reference standards of the studies used in this review leading to high heterogeneity. Also, another review by Ortiz *et al*. (2020) (24) reported high sensitivity and overall accuracy for the Vista Cam proxi in comparison with radiography which came in agreement with the results of the current study. However, there was a disagreement regarding the specificity values with the present study. This might result from the unclear risk of bias of most of studies included in the meta-analysis with exclusion the overlapped images from assessment which decreased the percentage of false results and increased the value of both sensitivity and specificity.

Following the analysis of this study results ,the null hypothesis- tested in this study-was partially accepted as there was a significant difference in the reliability of the near-infrared imaging system (VistaCam iX Proxi) when compared with ICDAS-II scores in detection of Proximal carious lesions while there was no significance difference for VistaCam iX Proxi when compared with digital radiographic findings.

## Conclusions

Under the conditions of the current study the following conclusions might be evident:

1-Near infrared transillumination based diagnostic modality is a promising method for detection of hidden proximal carious lesions overcoming the hazards of radiograph.

2-Near infrared transillumination is an accurate diagnostic method especially in detection of enamel lesions.
